# Teacher knowledge and teacher identity in mathematics education: An interdependent model

**DOI:** 10.12688/openreseurope.21108.1

**Published:** 2025-08-19

**Authors:** José Carlos Piñero Charlo, María del Carmen Canto López

**Affiliations:** 1 HUM462 – Desarrollo Profesional del Docente. Departamento de Didáctica, área de Didáctica de la Matemática, Universidad de Cádiz, Cádiz, 11510, Spain; 2 HUM634 - Psicología Lab. Departamento de Psicología, área de Psicología del Desallollo y la Educación, Universidad de Cádiz, Cádiz, 11510, Spain

**Keywords:** Professional identity; Professional profile; Mathematics education; Teacher training; Professional knowledge; Theoretical models

## Abstract

This article proposes an integrative theoretical model that articulates the interdependence between teacher professional knowledge and identity in mathematics education. Based on a systematic review of national and international approaches, it distinguishes the professional profile—defined by observable competencies and knowledge—from identity, which is constructed in a situated, dynamic, and reflective manner. The study draws on the MTSK model, Wenger’s theory of communities of practice, and the identity framework by Van Zoest and Bohl. It incorporates recent contributions on professional noticing, teacher beliefs, self-regulation, situational awareness, and context transfer. The resulting IPD model graphically represents this interdependence, offering a coherent framework for analysing teacher education pathways, professional decisions, and classroom practices. From a critical perspective, the article concludes that addressing identity and profile jointly enables the design of more relevant initial and continuing teacher education programs, as well as improvements in teacher evaluation and professional support systems.

## Plain language summary

This article offers a fresh perspective on what makes a great math teacher. We often focus on their "professional profile"—how much math they know, their teaching skills, and what can be observed and evaluated in the classroom. However, the article argues there's another crucial element: the teacher's "identity." This is deeper and more personal—how they see themselves as an educator, their values, their experiences, and how they evolve throughout their career. This identity isn't fixed; it's built dynamically through every lesson taught, every student interaction, and every professional development course.

Think of it this way: knowledge is the tools a carpenter uses, while identity is how they feel about their craft, their belief in their work, and their ability to adapt to new challenges. Both are deeply interconnected and constantly influence each other. You can't truly understand a teacher by looking at just one aspect.

The article introduces the IPD model to visually represent this interdependence. This model was developed by synthesizing extensive research on how teachers learn, their thought processes, classroom reactions, and how they apply their knowledge in different situations.

Ultimately, this understanding has practical implications: it can significantly improve teacher training programs, both for new educators and those with years of experience. By acknowledging that both a teacher's knowledge and their evolving identity are vital, we can design more effective training and support systems. The goal is to cultivate stronger teachers, leading to more engaging and meaningful math education for students.

Highlights •   Proposes an interdependent model linking teacher profile and identity.

•   Integrates MTSK, communities of practice, and identity frameworks.

•   Explains how beliefs and noticing shape teacher knowledge and self-perception.

•   Offers a framework to analyze and improve teacher education trajectories.

•   Addresses identity-profile tensions with practical, contextual implications.

## Introduction

In recent decades, there has been growing interest in understanding and conceptualizing the professional profile and identity of mathematics teachers. This interest stems from the need to improve teacher education, promote more effective teaching practices, and respond critically to contemporary educational challenges. In this context, the notion of “teacher profile” refers to the observable and classifiable characteristics of teachers—such as knowledge, competencies, and practices—while “teacher identity” refers to how teachers perceive and construct themselves professionally through interaction with their social and institutional environments.

Traditionally, these two constructs have been studied in parallel but separately: research on what teachers know and do has evolved independently from research on who teachers are and how they define themselves. This has resulted in a persistent methodological and conceptual fragmentation that hinders a comprehensive understanding of teacher development ( Lutovac & Kaasila, 2019). As a consequence, the field still lacks robust frameworks capable of integrating knowledge, beliefs, and identity in a coherent way that informs teacher education.

This article presents a review article of theoretical and critical nature, offering a synthetic and interpretative analysis of existing models of teacher knowledge and identity within mathematics education. Building on a comparative review of established frameworks—such as the Mathematics Teacher’s Specialized Knowledge (MTSK) model, communities of practice theory, and sociocultural approaches to identity—the article introduces a novel conceptual model that articulates both constructs in an interdependent structure. This review adopts an integrative lens that seeks to overcome conceptual fragmentation and contribute a coherent structure for guiding both research and educational practice.

Despite advances in both areas of study, several open questions remain: How does a teacher’s identity influence their professional decisions, such as curriculum emphasis or pedagogical choices? To what extent do formal qualifications, experience, or working context shape the development of professional identities? What kind of conceptual model can account for the mutual influence between identity and profile in teacher development?

Emerging studies have begun to highlight the reciprocal relationship between teachers’ identities and their professional knowledge. For instance, the emphases teachers place on disciplinary content, didactics, or reflective practice often align with their professional self-conception and are shaped by prior experiences ( Giadas *et al.*, 2024). Likewise, specific attributes of their professional profile—such as years of teaching or teacher education pathways—affect the ways in which identities are negotiated and redefined over time. However, in the absence of integrative models, it remains difficult to analyse transitions in teachers’ epistemological stances or to understand how particular conceptions about teaching translate into consistent patterns of professional behaviour.

Calls for more unified perspectives are increasingly present in the literature. Collaborative initiatives such as the MTSK international network have underscored the need to connect professional profiles with teachers’ belief systems and identities ( Giadas *et al.*, 2024). A unified approach would support the design of teacher education strategies better aligned with the real needs and trajectories of both prospective and practicing teachers.

As argued by Mandujano *et al.* (2025), identifying teacher profiles is not only a theoretical contribution but a foundation for developing differentiated and meaningful professional support. A simultaneous focus on profile and identity can inform a shared model of professionalism that enhances the effectiveness and consistency of teacher preparation. In educational systems increasingly driven by equity, innovation, and responsiveness—as in the Spanish context, among others—understanding teachers’ beliefs, competencies, and identities becomes essential. This understanding is necessary to design targeted interventions for pressing issues such as dropout prevention, gender equity in STEM, or the inclusion of migrant students.

In response to these challenges, the interdependent model presented in this article offers a conceptual contribution that reconceives teacher development as a dynamic, context-sensitive co-construction of knowledge and identity. By doing so, it aims to bridge existing gaps in the literature and provide a foundation for future research and pedagogical innovation.

## Methodological approach of the review

This article adopts a theoretical review methodology that combines a systematic and thematic approach to analyse and synthesize existing literature on mathematics teacher identity and teacher professional profiles. The aim of this review is not merely to summarize findings, but to construct a conceptual model that integrates both constructs in a mutually constitutive framework.

### Literature search strategy

The selection of sources followed a structured multi-stage process. First, a systematic search was conducted using the academic databases Scopus and Web of Science (WoS), which index peer-reviewed international journals with high standards of quality and visibility in educational research. The search was limited to articles published between 2015 and 2025, to ensure the relevance and currency of the theoretical perspectives included. Search terms included combinations of keywords such as: “teacher identity” AND “mathematics education”, “teacher profile” OR “professional knowledge”, “mathematics teacher” AND “beliefs” OR “epistemologies”, “didactics” OR “pedagogical content knowledge” AND “teacher development”. Additional sources were retrieved through backward snowballing (reviewing references cited in relevant articles) and forward citation tracking.

### Inclusion and exclusion criteria

To ensure conceptual and empirical coherence with the aims of the review, the following inclusion criteria were applied: (i) Articles published in peer-reviewed journals indexed in Scopus or WoS. (ii) Studies explicitly focused on mathematics teacher education, at primary, secondary, or university levels. (iii) Theoretical or empirical works that address at least one of the following: professional identity, teacher beliefs, professional profiles, or knowledge models in mathematics education. (iv) Publications written in English or Spanish, to reflect both international and Ibero-American research.

The exclusion criteria were: (i) Studies that addressed only student learning outcomes without focusing on teacher development. (ii) Generic studies on teacher identity that did not differentiate by disciplinary domain. (iii) Publications such as conference abstracts, editorials, or opinion pieces lacking peer review.

A total of 68 sources were initially identified, of which 43 were selected after applying the inclusion criteria and reviewing the abstracts and full texts. Among them, key frameworks and empirical findings were extracted, categorized thematically, and compared to identify complementarities, gaps, and tensions between models.

### Thematic synthesis

The selected studies were then subjected to a thematic analysis, focusing on how each addressed the dimensions of (a) professional knowledge, (b) identity construction, and (c) contextual mediation (e.g., institutional cultures, communities of practice). This thematic synthesis enabled the identification of structural disconnections between knowledge-based models (such as MTSK or MKT) and identity-based approaches (e.g., Wenger, Sfard & Prusak, Van Zoest & Bohl).

The review revealed that most studies treat knowledge and identity as separate research strands, which limits their applicability in designing comprehensive frameworks for teacher development. The proposed model in this article emerges from this gap, as a conceptual contribution that explicitly articulates the interdependence between epistemic and identity dimensions in teacher education.

### Limitations of the review methodology

While the review followed a structured process, some limitations must be acknowledged. First, the scope was restricted to peer-reviewed publications indexed in Scopus and Web of Science, potentially excluding relevant insights from grey literature or non-indexed regional studies. Second, although the review applied thematic coding to ensure analytical depth, the synthesis remains interpretative and influenced by the authors' theoretical positioning. Finally, the absence of a formal systematic review protocol may limit replicability, although transparency in criteria and selection procedures mitigates this concern.

## About professional knowledge

The study of mathematics teacher’s professional knowledge has gained international relevance in recent decades, particularly in the field of mathematics education. Research agrees that a solid professional profile requires the integration of disciplinary, didactic, and contextual knowledge, as well as situated cognitive skills. Several models highlight the importance of cognitive dispositions, situational competencies, and professional judgment in effective teaching performance. This section presents a reflection on and synthesis of the main conclusions on this topic.

### Professional knowledge of mathematics teachers

Professional teacher knowledge (here, TP) refers to the set of knowledge, skills, and dispositions that teachers use in their practice. It includes specific mathematical knowledge (content), pedagogical content knowledge, general pedagogical knowledge, and even knowledge of the educational context. This knowledge is currently conceived as multidimensional. In this regard, authors such as Blömeke *et al.* (2020) acknowledge the complexity of the teacher competence profile and the variability in emphasis: some teachers excel in content knowledge, while others in pedagogical skill.

Indeed, educational research has produced a consolidated theoretical corpus on teacher profiling, particularly within mathematics education. In the Spanish context, notable models include the MTSK model Carrillo Yáñez *et al.* (2022) and formulations inspired by the onto-semiotic approach Díaz Godino (2024), which richly describe the cognitive structure involved in didactic decisions.

However, while the separate treatment of teacher profile (TP) and teacher identity (TI) in mathematics has yielded valuable insights, focusing exclusively on professional knowledge tends to convey the idea that these are autonomous dimensions of teaching. This separation has crystallized in both methodological designs and resulting taxonomies, hindering analysis of how professional knowledge shapes identities—and vice versa. For instance, a teacher may demonstrate strong didactic content knowledge according to the MTSK model while holding a traditional conception of the teaching role, aligned with transmissive or reproductive identities. Similarly, literature on TI often focuses on teacher discourse or emotions without sufficiently considering how these positions relate to the conceptual frameworks guiding classroom decision-making.

### Constituent elements of the teacher’s professional knowledge

Since Shulman’s (1986) conceptualization of “teacher professional knowledge,” various models have been developed to organize mathematics teachers’ knowledge. Particularly relevant to this study is the MTSK model by Carrillo-Yañez *et al.* (2018) —based on Ball *et al.* (2008) model of Mathematical Knowledge for Teaching (MKT)—both for its theoretical strength and the consensus it has garnered regarding the essential knowledge for teaching mathematics. The MTSK model assumes that teachers’ knowledge is specialized and defines two broad domains: Mathematical Knowledge (MK) and Pedagogical Content Knowledge (PCK), each with more specific subdomains. A significant contribution of this model is the integration of teacher beliefs—about mathematics and its teaching—as an element permeating all subdomains: for instance, teachers’ beliefs about what mathematics is and how it is best learned shape their entire body of specialized knowledge.

**Figure 1.**
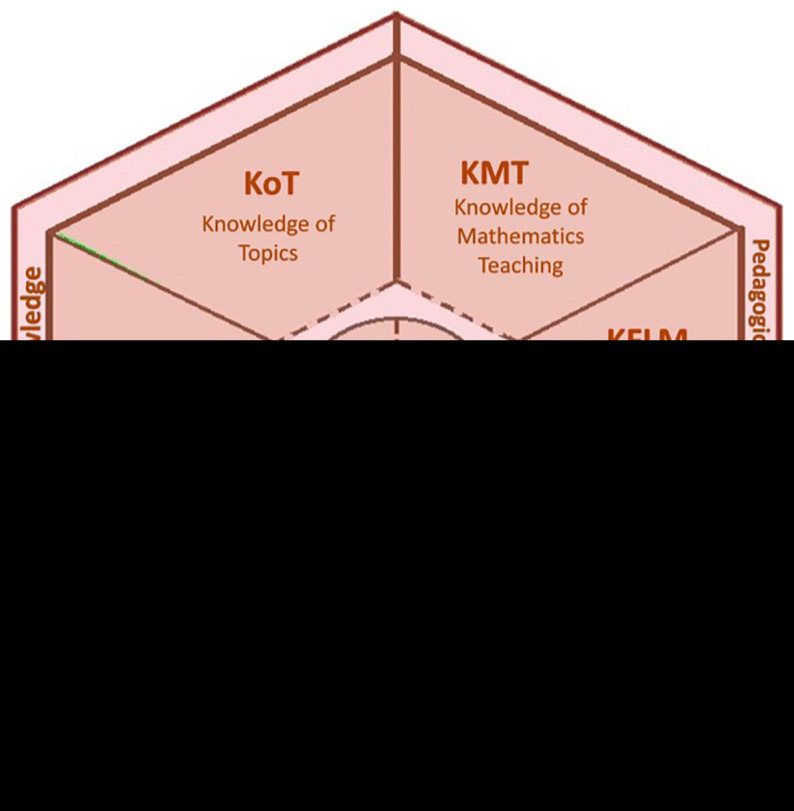
The Mathematics Teacher’s Specialised Knowledge model. From: Carrillo-Yañez *et al.* (2018).

A visual representation of the model’s components is shown in Figure 1 -from Aguilar-González *et al.* (2018)-. The Mathematical Knowledge domain includes understanding the connections between concepts, the structure of ideas, procedural reasoning, proofs, and different ways of engaging with mathematics, also considering mathematical language. Its subdomains are:

- Knowledge of Topics (KoT): a well-founded and deep understanding of mathematical content.
- Knowledge of the Structure of Mathematics (KSM): understanding connections between prior and later content, including internal structures of mathematics.
- Knowledge of Mathematical Practice (KPM): includes hierarchy and planning in problem-solving, validation and proof strategies, and the particular practices associated with mathematical tasks.

The model also retains the distinction between the MK and the Pedagogical Content Knowledge (PCK) domain. PCK includes the following subdomains:

- Knowledge of Mathematics Teaching (KMT): strategies to develop procedural and conceptual skills, use of representations, and selection of resources to support student understanding.
- Knowledge of Features of Learning Mathematics (KFLM): understanding student comprehension processes, concept-related language, and typical errors, difficulties, or misconceptions.
- Knowledge of Mathematical Learning Standards (KMLS): understanding what students are expected to achieve at each grade level, curriculum requirements, research findings, and expert opinions.

### Development of professional noticing and its relation to didactic knowledge

In mathematics education, professional noticing refers to a teacher’s ability to observe and interpret students’ mathematical thinking and respond accordingly. This concept involves three interrelated skills ( Jacobs *et al.*, 2010): (i) identifying significant mathematical strategies or elements in student work, (ii) interpreting the mathematical understanding evidenced by these strategies, and (iii) making instructional decisions to support learning. These skills define the competence of “noticing students’ mathematical thinking” and require not only attention to relevant aspects of classroom situations but also the ability to make sense of them through didactic knowledge and use that knowledge to improve teaching.

In Spanish context, several studies have examined how this professional noticing develops during teacher training ( Pérez-Montilla *et al.*, 2025). A recurring finding is that this competence relies heavily on the teacher’s didactic-mathematical knowledge, as only then can the teacher “observe in detail how students respond and approach problems” ( Weyers *et al.*, 2024; Zapatera Llinares & Callejo de la Vega, 2018). Proper interpretation of a student’s understanding also requires connecting observed details to broader mathematical concepts. In other words, professional noticing depends largely on specialized knowledge for teaching; as Ball *et al.* (2008) note, “teachers must activate their knowledge of mathematics teaching—both general and topic-specific—when interpreting students’ responses.”

Strong mathematical knowledge is thus necessary but not sufficient for expert noticing. This competence also demands explicit training and a reflective attitude. As Fernández *et al.* (2011) explain, “interpreting student productions is not an innate professional task—it can and should be developed through proper training.” The literature highlights that teacher knowledge and the use of that knowledge in practice are interdependent constructs Callejo and Zapatera (2017): knowing mathematics is important, but knowing how to apply it in classroom contexts—having an analytical view of learning situations—is equally crucial for effective teaching. Callejo and Zapatera (2017) identified progressive stages in the development of noticing competence: from students who cannot identify relevant mathematical elements in student work, to those who recognize them but cannot interpret student understanding, to those who interpret accurately and propose pedagogically appropriate responses based on that interpretation Llinares (2019). This evolution reflects a shift from a transmissive role to one of facilitator and investigator of students’ ideas, directly linked to the refinement of professional noticing.

It can be concluded that professional noticing is a fundamental construct for integrating the teacher’s competence profile and professional identity. On one hand, it encapsulates key competencies that define an effective teacher: pedagogical content knowledge, observation and analysis skills, and informed pedagogical decision-making. A teacher with strong noticing competence demonstrates a professional identity centered on student understanding, formative assessment, and continuous improvement. On the other hand, this competence both reflects and shapes teacher identity: those who adopt professional noticing see themselves as reflective educators focused on student learning. Teacher identity encompasses not only what the teacher knows but also how they view themselves in the role of learning facilitator ( Piñero, 2020). Developing noticing competence fosters teacher alignment with student-centered practices, reinforcing compatible beliefs and values—such as the idea that students’ errors are learning opportunities and a valuable source of insight into their thinking

## On teacher identity

International research has addressed mathematics teacher identity as a dynamic, situated, and narrative process. This implies that professional identity is constructed and reconstructed throughout a teacher’s life, integrating both personal and contextual experiences from teaching practice ( Naranjo Crespo, 2018). This section aims to reflect on and analyze the various approaches developed in this area.

### Teacher identity as a situated process

In the field of mathematics education, research has examined teacher professional identity using international theoretical frameworks adapted to local contexts. A widely used approach is the situated learning perspective, derived from the work of Lave and Wenger (1991). From this viewpoint, professional knowledge is not seen as an abstract entity possessed by the teacher, but rather as something constructed and expressed in social practice and intrinsically tied to the teacher’s identity. ( Wenger, 1998) notably argued that learning and identity are two sides of the same coin, stating that learning “is not simply about acquiring skills and information, but about becoming a different person.” In their original work, Lave and Wenger (1991) proposed that professional development occurs through participation in communities of practice, where peripheral participants gradually assume more central roles. In this process, these peripheral individuals forge their identity as competent members of the group. Applied to mathematics education, this means that one learns to become a mathematics teacher not merely by acquiring knowledge, but by participating in the practices, discourses, and values shared by the mathematics teaching community.

In mathematics education specifically, Hodgen (2011) proposed a "situated theory of mathematical knowledge for teaching," arguing that teacher knowledge is inherently situated in practice and inseparably linked to identity as a participant in those practices. In this approach, concepts such as the teacher’s professional narrative or classroom discourse participation become as relevant as declarative knowledge, as they reflect how the teacher sees themselves (identity) enacting their professional knowledge in real contexts. More recently, mathematics education research has used the situated perspective to interpret teacher development. For example, Graven and Lerman (2014), in a study with in-service mathematics teachers, emphasized the central importance of "confidence" in identity construction. Their findings show that as teachers gained mastery of new practices and content, their confidence increased, and their self-image evolved toward that of educators. This increase in confidence was interpreted as an essential component in the construction of emerging professional identity, intimately linked to learning achieved in community.

Moreover, variables such as experience and level of training shape opportunities for developing specific identities. A recent study on mathematics education assessment identified two distinct teacher profiles based on the frequency with which they implement different evaluative practices, revealing that factors such as years of experience, participation in continuing education, and school context—although with a small effect—differentiate between the profiles Mandujano *et al.* (2025). This suggests that a teacher’s professional trajectory influences how they conceptualize and enact their practice, reflecting distinct identity stances toward assessment.

It may be concluded that these sociocultural approaches are useful for studying how teachers negotiate their professional identity when adopting pedagogical innovations, or how peer learning communities function as spaces for knowledge construction and belonging. Available evidence shows that when mathematics teachers engage in reflective communities (such as lesson study groups, study collectives, or professional networks), processes of co-construction of knowledge and identity take place: teachers assimilate new didactic-mathematical knowledge while also gradually adopting the values, discourse styles, and attitudes of the community—in short, they develop their identity as “good mathematics teachers” within that group. Theorists like Wenger describe this process by stating that identity “emerges from personal knowledge and its alignment through participation in the community.”

### Processes of constructing teacher profile and identity

Studies such as those by Bezerra De Melo *et al.* (2024) show that educators of future mathematics teachers—since they are agents of professional socialization—have a direct impact on the identity construction of their students. This influence is exerted explicitly through the discourse and practice of teacher educators, and also implicitly via what Ronca (2007), as cited in Bezerra De Melo *et al.* (2024), refers to as "internalized models." Thus, the future mathematics teacher’s identity construction is also conditioned by the identity of their instructor.

However, the construction of TP and TI does not conclude with initial teacher training; it continues throughout the professional career. In Spain, institutions and professional networks offer courses, conferences, and working groups to update knowledge and share best practices. Nevertheless, structural obstacles persist ( Piñero, 2020), as—despite the widespread discourse emphasizing the importance of innovation and teaching research—real conditions for engaging in such activities in daily school life are lacking, leaving ongoing training as an almost exclusively personal responsibility. Moreover, studies like those by Llinares and Callejo de la Vega (2018), conclude that mastery of mathematics is “mandatory but not enough” for developing an adequate “professional noticing” of student learning. In other words, teacher identity in mathematics is configured through the integration of mathematical knowledge, didactic expertise, and competencies related to noticing or interpreting students’ understanding. This reflects the traditional tension between disciplinary and pedagogical training, while also introducing a third essential factor.

Summarizing, mathematics teacher professional identity is currently understood as a situated, dynamic, and continuously evolving process, shaped by personal, social, and professional experiences. Indeed, research in mathematics education ( Contreras *et al.*, 2011) emphasizes that teachers build their identity in response to questions like “Who am I as a teacher at this moment, and who do I want to become?”—redefining their role as they gain experience. These studies affirm that while mathematical mastery is essential, it is not sufficient for developing a professional perspective that is attentive to student learning.

## Cognitive factors

A model that aims to recognize interdependence must assume that certain cognitive factors of the teacher profile (knowledge, beliefs, reasoning skills, etc.) are closely linked to how the teacher perceives themselves in their professional role (identity). This section presents and analyzes the main cognitive factors to be considered in the model.

### Beliefs about mathematics and its teaching/learning

Beliefs about mathematics, its teaching, and its learning constitute interpretive frameworks that guide practice (sometimes even stronger than formal knowledge). These beliefs act as filters through which teachers interpret classroom situations and define their role ( Azcárate Goded, 2001). For instance, a teacher who believes that “mathematics is only for a few” will tend to generate selective practices, while one who believes that all students can learn will be more inclined to adapt teaching to student diversity. In this regard, two clear categories can be established—based on NCTM principles: productive and unproductive beliefs ( Leinwand *et al.*, 2014).

### Beliefs about mathematics and its teaching/learning

Professional self-regulation refers to a teacher’s ability to reflect on their own practice, identify areas for improvement, and take formative actions toward continuous professional growth. It encompasses metacognitive skills such as self-assessment, planning for improvement, and managing emotions and motivations in teaching. In building professional identity, self-regulation plays a key role: teachers who see themselves as “lifelong learners” adopt an evolving identity, open to change and development. Literature highlights the importance of reflection and inquiry into one’s own practice as drivers of identity development. Beijaard *et al.* (2004) for example, emphasize that reflection on teaching experiences and self-assessment as a professional are factors that strengthen teacher identity by articulating theory with practice and aligning actions with personal beliefs and values. Encouraging self-regulation during initial teacher education involves guiding future teachers to become aware of their progress and difficulties, which translates into promoting action research, collaboration in teaching communities, and ongoing professional development.

### Student perception

The development of professional noticing requires the coordination of didactic knowledge, sensitivity to diverse strategies, and readiness to adapt to practice in real time. Mathematics learning, as a form of social interaction, requires understanding not only what another person says, but also why and how they say it—in order to anticipate their intentions, reactions, or emotional state. This factor involves the teacher’s ability to attribute mental states to students and to anticipate their comprehension processes or potential difficulties. This ability is not merely empathetic, but deeply analytical and situated, requiring the teacher to operate with complex internal representations of how their students learn. The effective activation of this competence—visible in teachers who “read between the lines” of students’ responses—implies an identity commitment: teachers who see themselves as facilitators of thinking tend to develop a more attentive, patient, and diagnostic gaze

### Situational awareness and decision-making

Another key set of cognitive factors is the teacher’s ability to act with professional judgment in the complex environment of the mathematics classroom. It is well-documented that mathematical classroom discussions and debates contribute to learning progress, as shown in the works of Bishop (1972; 2008), among others. However, such debates, which require reasoning, activate high cognitive demand conditions: the classroom is a dynamic environment, saturated with stimuli, where attention must be constantly distributed and updated. Hence, the importance of situational awareness—the teacher’s ability to perceive, interpret, and anticipate what is happening in the classroom in real time. This awareness is not a form of passive observation; rather, it is an active process of meaning-making that enables the teacher to respond with flexibility and relevance.

## Interdependence between teacher profile and identity

The discussion presented in this document on teacher profiles and identities in mathematics education is structured by three key elements: professional teacher knowledge, teacher identity, and cognitive factors involved in the development of “professional noticing.” This section outlines a model that addresses the interdependence between teacher profile and identity (IPD model)

### Integrative models

Regarding the study and construction of integrative frameworks for teacher profile (TP) and teacher identity (TI), studies such as Lutovac and Kaasila (2019) call for the inclusion of mixed approaches and emphasize the need for a model that articulates diverse theoretical and methodological perspectives. A pioneering model that directly integrates teacher identity and professional knowledge in mathematics education is that of Van Zoest and Bohl (2005), who developed a theoretical framework to study how secondary teachers learn to teach mathematics, simultaneously considering what they know and who they become as teachers. This framework combines three key constructs into an integrative model in which mathematics teacher identity is understood as the intersection of didactic-mathematical knowledge and participation in professional communities:

- Shulman’s (1986) heuristic on essential knowledge for mathematics teaching (disciplinary, curricular, and pedagogical knowledge expected of an effective teacher).
- Wenger’s (2001) conception of learning as identity development through participation in communities of practice.
- Cognitive notions that recognize teachers’ individual mental processes.

In their model, Van Zoest and Bohl introduce two major interrelated components: the “self-in-mind” and the “self-in-community.” The self-in-mind aspects refer to what the teacher possesses individually at the cognitive level: mathematical content knowledge, pedagogical content knowledge, beliefs, attitudes, intentions, and personal commitments to teaching. The self-in-community components recognize the social facet of teacher identity: how the teacher participates in professional communities (e.g., math departments, collaborative groups, professional networks), how they are perceived by others, how they perceive others’ views of them, and their alignment with the norms and values of the educational community.

**Figure 2.**
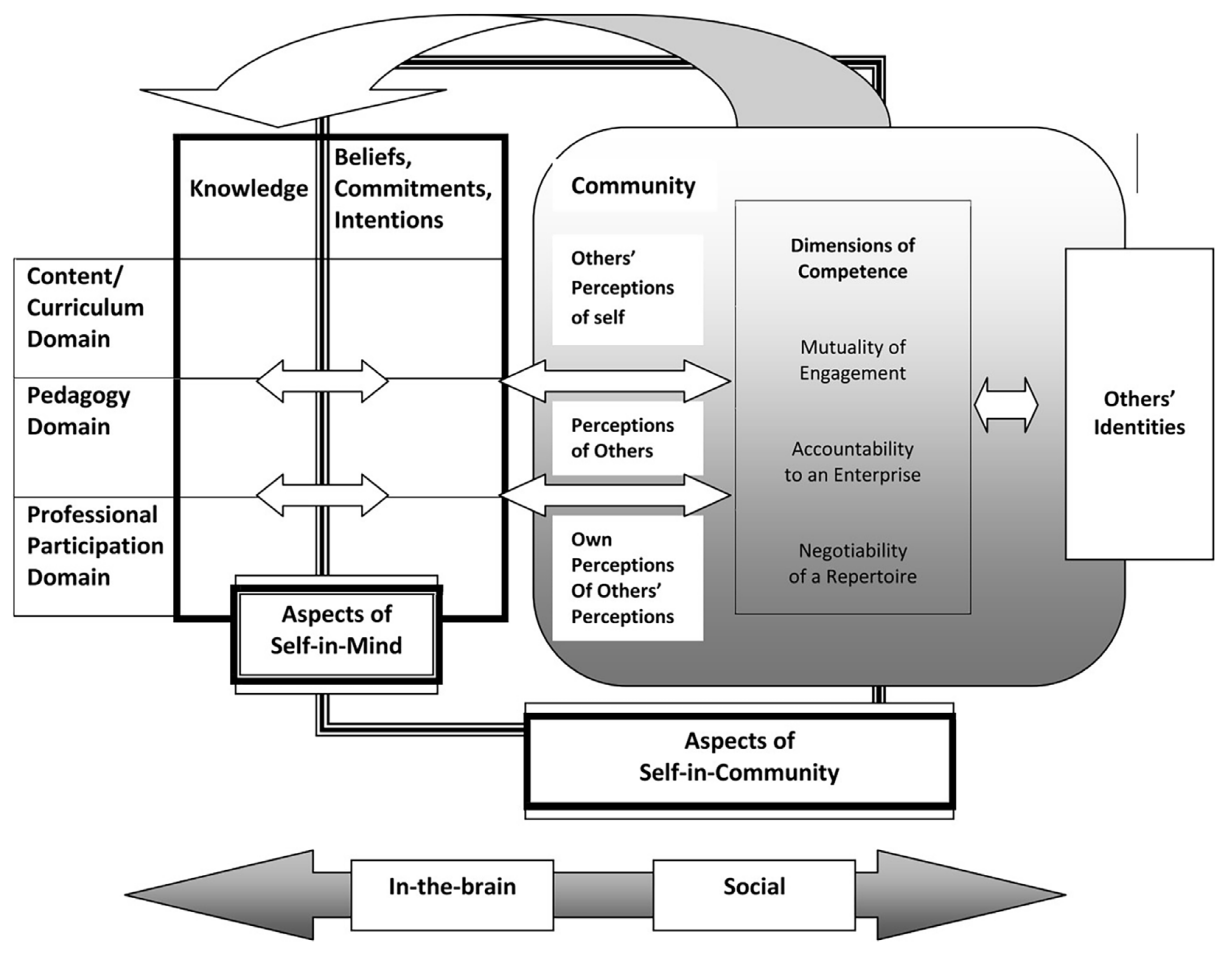
Mathematics Teacher Identity framework. From: Sun (2017).

A central element of this model is that professional development occurs through the interaction of these aspects. Teachers build their identity by adopting roles and competencies within the community while simultaneously constructing internal knowledge and beliefs. Van Zoest and Bohl emphasize that teacher learning and knowledge exist on a continuum—from individual to social—that includes all intermediate stages -see Figure 2, from ( Sun, 2017)-. Within this framework, teacher learning is understood as neither purely internal nor solely social; it is “not just an accumulation of skills and information, but a process of identity development through social interactions.” Embracing this idea implies recognizing that changing the way one teaches mathematics involves both acquiring new knowledge and reconfiguring one’s teacher identity through interaction with others. The model incorporates several dimensions in the construction of identity:

- Mutuality of engagement, developed directly in the classroom community with students or through interactions with peers.
- Accountability to an enterprise, reflecting adherence to the standards and norms of an institution, often enforced through internal evaluations or expectations.
- Negotiability of a repertoire, involving participation in and co-ownership of the community’s shared practices, tools, and knowledge developed over time to achieve its goals.

One implication of this model is that a strong teacher identity tends to be associated with greater content and pedagogical knowledge, as well as more active participation in professional communities. For professional development, this framework suggests that training should address both dimensions: providing new knowledge and opportunities for practice, while also supporting teachers in reconstructing their identity—including beliefs, confidence, and sense of professional belonging. Thus, the Van Zoest and Bohl model offers a holistic conceptual framework for studying the development of mathematics teachers by integrating the cognitive and social dimensions of their growth.

### From conceptual analysis to model construction

The previous sections have examined a range of theoretical frameworks related to teacher knowledge and identity in mathematics education. While these models offer valuable insights, they tend to treat teacher profile and identity as distinct domains, often resulting in parallel yet disconnected analyses. This separation limits our ability to capture the full complexity of teacher development as a dynamic and situated process. To address this, we move beyond the juxtaposition of conceptual models and propose an integrated framework: the Interdependent Profile-Identity (IPD) model.

To support the model’s design, a comparative synthesis of key frameworks was conducted, focusing on how each conceptualization addresses three fundamental dimensions: (a) the epistemic dimension (knowledge and cognition), (b) the relational dimension (self-perception and positioning), and (c) the contextual dimension (institutional and sociocultural influences). Table 1 summarizes this synthesis, highlighting convergences and gaps among selected models.

**Table 1.** Comparative mapping of key frameworks across identity-profile dimensions.

Framework/Model	Epistemic (knowledge)	Relational (identity)	Contextual (positioning)
MTSK ( Carrillo-Yañez *et al.*, 2018)	Deep content and pedagogy	Not addressed	Limited
MKT ( Ball *et al.*, 2008)	Pedagogical knowledge	Not addressed	Not explicit
Communities of Practice ( Wenger, 1998)	Not knowledge-focused	Practice-linked identity	Strong situational focus
Identity positions ( Van Zoest & Bohl, 2005)	Implicit	Typology of identity roles	Situated in practice contexts
Sfard & Prusak (2005)	Not specified	Narrative identity	Emphasis on discourse and roles

This comparative analysis reveals an asymmetry: while some models (e.g., MTSK, MKT) provide robust accounts of professional knowledge, they neglect how this knowledge connects to the teacher's sense of self or role within a community. Conversely, identity-oriented frameworks (e.g., Wenger; Sfard & Prusak) focus on narrative, social, or relational aspects of teacher identity but lack an operationalization of professional knowledge structures.

The design of the IPD model thus emerges from the need to reconcile these dimensions. Its architecture is organized into three interrelated subdomains:

- Knowledge and Beliefs (Epistemic Core): comprising both content-specific knowledge and pedagogical reasoning, informed by MTSK and enriched with notions such as professional noticing and reflective judgment.
- Identity Configuration (Relational Layer): incorporating narrative positioning, perceived agency, and emotional self-understanding, based on identity theory and discourse-based models.
- Situated Membership (Contextual Interface): including institutional norms, community participation, recognition processes, and social expectations, grounded in practice-based and sociocultural theories.

These subdomains are not conceived as separate layers but as interacting spheres that co-construct the teacher’s professional development over time. Their interdependence supports the hypothesis that changes in one dimension (e.g., identity reconfiguration) will affect the others (e.g., uptake of professional knowledge or pedagogical stance).

### Interdependent model of mathematics teacher Profile and Identity (IPD)

Recent findings, such as those published by Bezerra De Melo *et al.* (2024), support the need to adopt an interdependent approach to analyzing TP and TI, particularly within mathematics teacher education. Their study demonstrates that identity construction processes do not occur in isolation, but rather emerge from a complex dialectic among personal, professional, academic, and disciplinary factors. In this view, mathematics teacher professional identity (IPD) is conceived as a dynamic construction that integrates family and social backgrounds, academic trajectories, the influence of previous teachers, and—critically—the teacher’s relationship with mathematics and mathematics education as fields of knowledge. Bezerra and colleagues conclude that IPD cannot be analyzed as a closed category or merely as a self-image but is deeply intertwined with the practices and knowledge that comprise the teacher’s profile.

A framework aiming to recognize IPD interdependence must uphold the central thesis that the professional teacher profile cannot be defined exclusively by observable competencies or functional traits but must instead be understood as the outward projection of an identity process in constant evolution. Likewise, teacher identity is shaped and redefined in response to the profile the teacher expects to assume—or is expected (or pressured) to assume—by the institution. Based on the sources discussed, an interdependent IPD model can be synthesized around the following categories:

- The distinctive elements of the mathematics teacher’s professional profile, particularly those identified within the MTSK framework ( Carrillo-Yañez *et al.*, 2018).
- The development of teacher identity as a situated process ( Hodgen, 2011).
- The cognitive aspects, especially those related to the development of professional noticing ( Jacobs *et al.*, 2010).

The construction of this model assumes that teacher identity is the product of situated practice. Studies such as those by Chávez and Llinares (2012) indicate that teachers negotiate their identity through daily classroom participation. These authors offer empirical support for the sociocultural claim that identity emerges from engagement in social practices—professional practice thus acts as both the setting and catalyst for identity development.

**Figure 3.**
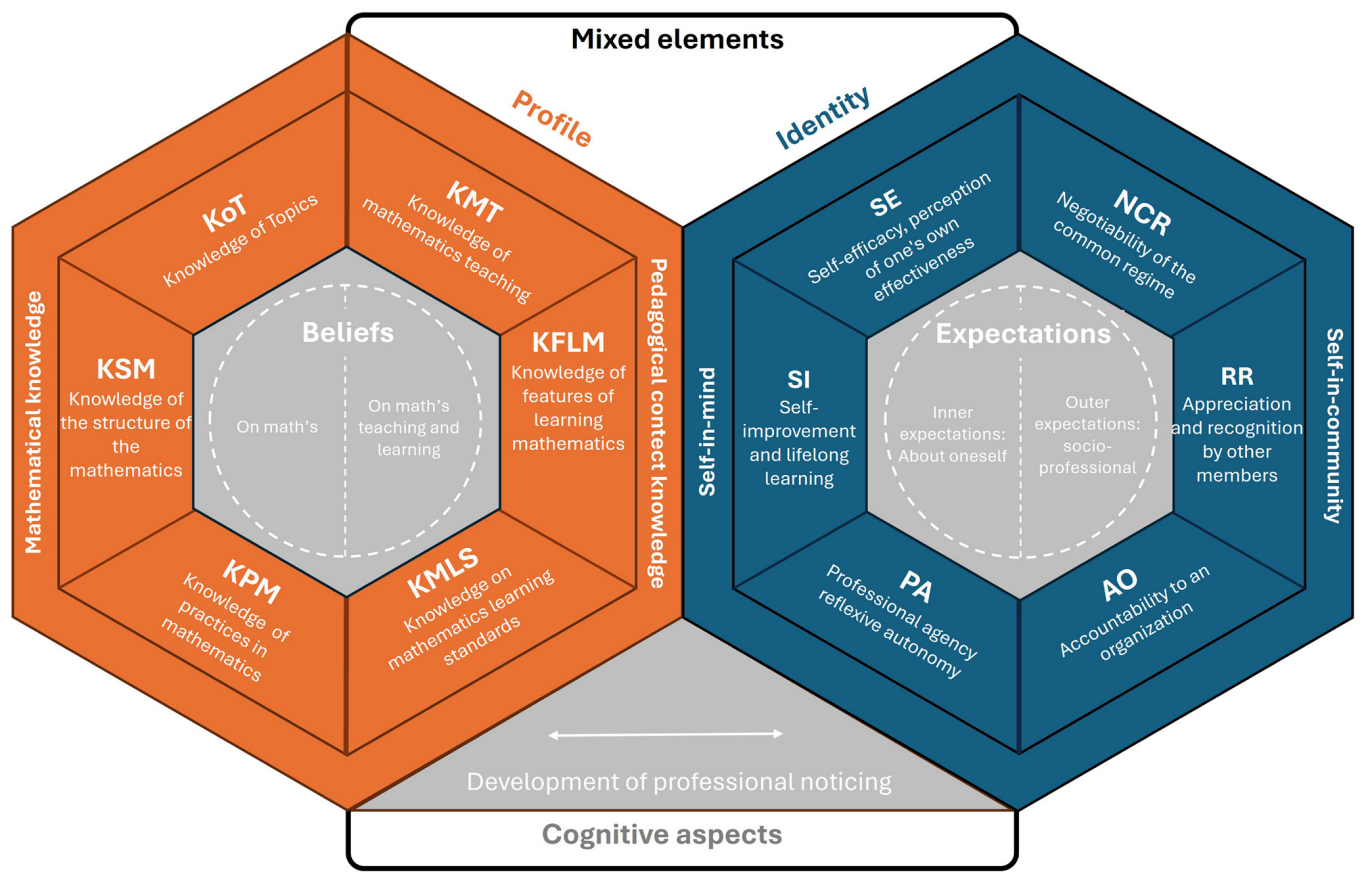
IPD framework.

Additionally, the development of professional noticing is inherently interdependent, grounded in cognitive elements and concrete aspects such as reflective capacity and student perception. In this regard, research presented at SEIEM, such as that of ( Contreras *et al.*, 2011), shows that preservice teachers, when reflecting on their past experiences as students and comparing them with their current training, identify tensions and continuities that shape their identity, for example, rejecting traditional rote methods and aspiring to become more dynamic teachers. These and other contributions add an important nuance to the sociocultural approach: while identity is socially constructed, self-reflection is the tool through which individuals assimilate and internalize social influences, giving coherence to their own professional biography. Consequently, the proposed model (see Figure 3) considers a broad set of cross-connections and interdependencies.

### Structure of the model

The interdependent IPD model proposed here is organized around three main pillars: the aspects of the “self-in-mind,” the “self-in-community,” and the mixed elements that mediate between them. This structure revisits and updates the theoretical framework of Van Zoest and Bohl (2005), who conceptualized teacher identity as the result of the interaction between individual cognition and community participation. Building on that foundation, categories from the MTSK model are integrated to characterize the teacher’s professional profile, and the construct of “professional noticing” is incorporated as a key axis to examine the connection between practice and identity.

The professional component (teacher profile) of the model employs the categories from the MTSK framework and its developmental philosophy to distinguish two internal domains: the “self-in-mind” and the “self-in-community.” It is composed of the following subdomains:

- Self-efficacy (SE): defined as the teacher’s perception of their own ability to teach and their mastery of the professional knowledge and skills they believe they possess. It is thus a judgment about one’s own capacity.
- Self-improvement (SI): reflects the individual’s commitment to continuous improvement, training and lifelong learning. It indicates the extent to which one adopts a growth mindset that enables Professional Agency.
- Professional Agency (PA): this subdomain reflects the extent to which the teacher feels responsible and autonomous in transforming their own practice. It refers to the will, decision-making, and responsibility to act upon oneself (and one’s professional environment).

While the concepts of self-efficacy and self-training are well-established in mathematics education—and are included in validated questionnaires for characterizing teacher identity, such as the MTEBI ( Enochs *et al.*, 2000; Segarra & Julià, 2022)—Professional Agency is defined in the literature ( Gardee, 2019) as the teacher’s capacity and willingness to implement transformative agency in the classroom. Such transformative practice affects both the students’ mathematical identity and the teacher’s own.

Meanwhile, the aspects of situated identity are considered within the “self-in-community” component, which encompasses the social, contextual, and biographical factors that shape teacher identity. These include participation in educational communities, the teacher’s self-perception, and how they are perceived by others. This dimension recovers the notion of identity as a situated and negotiated process built through interaction ( Hodgen, 2011), in which shared practices and discourses shape not only what the teacher does, but who they are as a professional. It incorporates key elements in identity construction—educational background, school experiences, shared values, professional networks—as factors directly influencing identity formation. Thus, the “self-in-community” domain represents the relational, situated, and socially constructed dimension of professional identity. It includes three subdomains:

- Negotiability of the Common Regime (NCR): refers to the teacher’s ability to interpret, question, appropriate, and modify—explicitly or implicitly—the norms, practices, and shared meanings that shape their professional community.
- Relational Recognition (RR): refers to the extent to which the teacher is recognized, valued, and legitimized by other members of the professional community (peers, students, families, institution) as an active, competent, and authorized participant. This recognition not only contributes to the sense of belonging but directly influences identity formation.
- Accountability to an Organization (AO): refers to the way in which the teacher assumes responsibility to external entities that regulate, assess, or supervise their professional practice. This subdomain encompasses both formal obligations to comply with curricular standards, evaluation policies, or institutional norms, and the ethical and professional disposition to respond to these expectations reflectively and critically.

The RR subdomain complements NCR and AO by introducing the intersubjective dimension—how identity is also constructed externally. It connects to the emotional component of recognition, as feeling valued by others reinforces one’s commitment to the community. Moreover, RR has clear practical implications: a teacher who does not perceive themselves as recognized or included tends to withdraw, reduce participation, or abandon professional leadership.

### Inner dialogue and possibilities

The IPD model presents a robust and well-balanced conceptual architecture, filled with internal connections worth highlighting. In this regard, expectations form the central organizing element of the model’s “identity” dimension. Teachers’ expectations guide their judgments of self-efficacy (SE), their decision to pursue professional learning (SI), and their willingness to act (PA). Simultaneously, external expectations shape the context in which the teacher is held accountable (AO), seeks recognition (RR), and negotiates meanings (NCR). The model thus recognizes how teachers accept, reject, or reinterpret these expectations—and the impact this has on their identity. By placing expectations at the core of the identity dimension—acting as a sort of “semi-permeable membrane” between the “self-in-mind” and the “self-in-community” domains—the model allows for the integration or filtering of social mandates in accordance with the teacher’s values, knowledge, and aspirations.

However, the model’s interconnections extend beyond this, as every potential crossover within the “identity” dimension can be explored. While not all intersections will be discussed here, a few are worth noting:

- SI–AO: The commitment to improvement may be driven by external demands that the teacher transforms into personal goals. Lifelong learning thus becomes an ethical response to institutional and social demands for professional quality.
- SE–RR: Perceived personal competence is reinforced or undermined by the recognition received from others. An environment that validates a teacher’s knowledge and actions enhances self-efficacy; one that discredits them may erode it—even if the teacher has solid training.
- PA–NCR: Agency is externally manifested in the ability to influence collective norms. A teacher exercising transformative agency tends to actively participate in the construction and renegotiation of the shared repertoire.

Additional interconnections can also be explored within each domain. In the “self-in-mind” domain, for instance, the following relationships can be observed:

- SE–SI: A strong self-efficacy judgment promotes a commitment to lifelong learning by reinforcing the belief that training efforts will yield results. Conversely, commitment to professional development may enhance self-efficacy by providing new tools, strategies, and successful experiences.
- SI–PA: Self-training is a key enabler of agency. Teachers who take responsibility for their own growth are better positioned to act autonomously, make transformative decisions, and innovate in their practice.
- SE–PA: Agency is mediated by perceptions of competence: a teacher who doubts their knowledge or skills is unlikely to act agentively. At the same time, acting successfully reinforces self-efficacy in a virtuous cycle.

Beyond these internal mechanisms, the model offers several practical possibilities for analysis. One such use is identifying identity tensions that arise when subdomains from different areas are misaligned or in conflict. For example, a teacher with strong professional agency (PA) and commitment to learning (SI) may feel frustrated or alienated if their professional environment limits participation in renegotiating the shared repertoire (NCR) or fails to provide adequate relational recognition (RR). This tension between self-image and contextual conditions may lead to identity redefinition, withdrawal, or active resistance, depending on how internal and social-professional expectations are managed.

Secondly, the model enables descriptions of identity development processes through the progressive strengthening of subdomains. For instance, the trajectory from emerging self-efficacy to consolidated professional agency could be understood as a path of identity maturation—one involving not just increased perceived competence but also deeper transformative engagement. Likewise, a teacher’s gradual integration into the shared repertoire—from passive compliance to active participation—can be seen as a process of professional community appropriation.

Finally, the model provides a tool for mapping diverse identity trajectories, understood as specific combinations of subdomains that shape distinct identity profiles. For example, a teacher with high relational recognition (RR) but low professional agency (PA) may adopt a more conformist identity, while one with strong self-training (SI) and openness to negotiating the shared repertoire (NCR) may embody an innovative, transformative identity. These trajectories are not fixed, but subject to change—reinforcing the idea of identity as a situated and continuously evolving process.

## Conclusions

This paper has explored existing literature with the aim of investigating and synthesizing the co-determination between the teacher profile and teacher identity in mathematics education, in order to integrate both within a common framework that better captures the complexity of teaching practice and promotes professional growth in a comprehensive manner. In this regard, the paper discusses the transformation that the characterization of the teacher profile has undergone in recent decades: from early work focused on content knowledge and pedagogy ( Carrillo-Yañez *et al.*, 2018; Shulman, 1986) to more integrative approaches that simultaneously address cognitive, affective, contextual, and social dimensions ( Blömeke *et al.*, 2020; Lutovac & Kaasila, 2019). These studies are framed within an international research landscape that has also examined the relationship between teacher profiles and other elements such as professional identity ( Beijaard *et al.*, 2004), instructional quality, assessment ( Mandujano *et al.*, 2025), and learning opportunities. However, a persistent question in the literature—which this paper seeks to address—remains: how can cognitive, professional, identity, and discursive dimensions of the teacher be articulated within a single model?

Drawing on a review of existing theoretical frameworks, this paper proposes a model that seeks to overcome the dichotomy between professional profile and identity, integrating them within a unified analytic matrix. This matrix aims to describe mathematics teachers’ training trajectories, understand their professional decisions and career paths, and most importantly, design teacher education programs that take into account not only observable aspects of teaching performance, but also the subjective and relational components that shape what it means to be a teacher.

This perspective is especially relevant in contexts where tensions persist between disciplinary training and pedagogical preparation, and where professional recognition of university teaching has yet to be systematically linked to reflection on identity. In this sense, the model proposed here is not merely a theoretical framework, but also a tool to rethink teacher education programs and quality assessment systems, aligned with a more humanized, situated, and transformative vision of mathematics education. The framework presented contributes to ongoing discussions about the complex interrelationship between identity and teacher profile, acknowledging the inherent limitations of constructs that are, by nature, constantly evolving. Thus, the model is intended to be more structural than merely descriptive, in order to facilitate its operationalization in training contexts.

The model presented in this article responds to a critical gap: it proposes a conceptual structure in which teacher profile and professional identity are not parallel dimensions, but mutually constitutive. In contrast to existing models that tend to analyze “knowing how to teach” and “being a teacher” separately, the interdependent proposal put forth here builds on the MTSK framework and expands it to show how professional knowledge interacts with the construction of identity meanings—and how particular identity configurations influence the appropriation and development of such knowledge.

Taken as a whole, the model not only allows for the analysis of teacher identity as a structural construct, but also as a dynamic field of possibilities, shaped by tensions, interactions, and shifts that define the professional development of mathematics teachers.

On the other hand, while the IPD model offers a comprehensive framework for understanding teacher development, it is not without limitations. Its emphasis on reflective practice, identity negotiation, and situated knowledge may render it less applicable in highly prescriptive educational systems where teacher autonomy is limited and professional roles are tightly standardized. In such contexts—often characterized by rigid curricula, centralized accountability, or limited opportunities for teacher agency—the dynamic interplay between identity and knowledge may be constrained, reducing the explanatory power of the model. Furthermore, the model assumes access to collaborative and dialogic professional environments, which may not be present in all institutional settings.

Finally, it is worth noting that among the many challenges identified, one of the most salient is the coexistence of multiple profiles within the same teaching staff. This difficulty also presents an opportunity to explore the unique potential and limitations of each profile ( Giadas *et al.*, 2024) and implies recognizing that there is no single model of what it means to be a teacher, but rather multiple ways of being one, each associated with distinct professional identities.

## Ethics and consent

Ethical approval and consent were not required.

## Data Availability

No data are associated with this article.
