# Supplementary material for: Teacher knowledge and teacher identity in mathematics education: An interdependent model
Source: Open Res Eur. 2026 Apr 16;5:246. Originally published 2025 Aug 19. [Version 3] doi: 10.12688/openreseurope.21108.3 (PMC13032108; doi:10.12688/openreseurope.21108.3)
Supplement: Peer review history

# Reviewer report: Reviewer response for version 2

**DOI:** 10.21956/openreseurope.24907.r70028

**Published:** 2026-03-27

**Authors:** Jorge Guerra Antequera

**Affiliations:** 1 University of Extremadura, Cáceres, Spain

**Summary of the Manuscript:** this article introduces the Professional Identity and Knowledge (PIK) model, designed to link the historical gap between teacher knowledge (the "profile") and teacher identity in mathematics education. By synthesizing established frameworks; specifically the Mathematics Teacher’s Specialized Knowledge and Wenger’s Communities of Practice. Author argue that professional development is not merely an accumulation of cognitive competencies but a dynamic, situated process of "becoming." The model identifies key psychological and cognitive bridges, such as beliefs and professional noticing, which facilitate the interdependence between what a teacher knows and who a teacher is.

**Critical Evaluation and Recommendations:** the manuscript provides a strong  theoretical contribution; however, certain areas require further clarification to ensure scientific rigor:

**1. Methodological Transparency:** While the authors frame the study as an integrative synthesis, the transition from the literature review (43 sources) to the specific geometric structure of the PIK model should be more explicitly detailed. Providing a brief description of the thematic coding process would strengthen the validity of the proposed framework.

**2. Operationalizing Interdependence:** To move beyond abstraction, the authors should provide concrete examples of how specific knowledge domains (e.g., Knowledge of Topics) influence identity narratives in practice. This would clarify the "bidirectionality" that the model claims to represent.

**3. Practical Application:** The manuscript would benefit from discussing how the PIK model can be operationalized as a diagnostic tool within teacher education programs.

**Conclusion:** the PIK model is a timely and significant advancement for the field. If the authors address these minor methodological and justificatory points, the article will serve as a foundational resource for understanding the holistic development of mathematics educators. The language is sophisticated and appropriate for an international academic audience.

Are all factual statements correct and adequately supported by citations?

Yes

Is the review written in accessible language?

Yes

Are the conclusions drawn appropriate in the context of the current research literature?

Yes

Is the topic of the review discussed comprehensively in the context of the current literature?

Yes

Reviewer Expertise:

Educational technology, Videogames and education, VIdeogames and culture, videogames and Mathematics

I confirm that I have read this submission and believe that I have an appropriate level of expertise to confirm that it is of an acceptable scientific standard.

# Response

**Published:** 2026-03-29

**Authors:** José Carlos Piñero Charlo

Dear Reviewer, Prof. Jorge Guerra: We sincerely thank you for the time and care devoted to reading our manuscript and for your constructive and encouraging assessment. We particularly appreciate your recognition of the PIK model as a timely theoretical contribution and of the manuscript’s overall scientific standard. Below, we respond point-by-point to your recommendations, indicating where the revisions were implemented in the revised manuscript.

**1. Methodological transparency: clarifying the transition from the corpus to the geometric structure of the PIK model** We fully agree that the transition from the reviewed literature (43 sources) to the model’s specific structure needed to be more explicit. In response, we strengthened the methodological account by adding a dedicated subsection describing the thematic coding workflow and how it informed model construction.

- **Added a detailed “Thematic coding and model construction” subsection (Section 2.3**): We now specify the unit of analysis (“conceptual claims”), the two-cycle coding process, the role of iterative refinement, and the explicit transition from thematic coding to relational modelling.
- **Added and referenced a flow diagram (Figure 1): **Figure 1 summarizes the coding and modelling workflow visually, making the methodological progression from corpus delimitation to relational modelling transparent.

These additions were intended to strengthen the validity and transparency of the proposed framework by showing, step-by-step, how thematic coding outcomes were reorganised into the model’s relational architecture rather than presenting the geometry as a purely authorial design choice.

**2. Operationalising interdependence: providing concrete examples of bidirectionality** We agree that “bidirectionality” benefits from being illustrated through concrete, practice-grounded examples that clarify how knowledge domains and identity narratives can co-evolve.

- **Added a new subsection “Illustrative bidirectional links in practice” (Section 6.7):** This subsection provides two short scenarios to exemplify (i) how Knowledge of Topics (KoT) may contribute to identity narratives/positionings via bridging constructs (e.g., professional noticing and epistemic orientations), and (ii) how identity narratives/positionings may shape the activation and prioritisation of knowledge resources via bridging constructs (e.g., self-regulation and situational awareness).

We also explicitly frame these scenarios as conceptual exemplifications rather than empirical cases, in line with the integrative-theoretical nature of the review, while still making the proposed interdependence more concrete and interpretable.

**3. Practical application: operationalising PIK as a diagnostic tool in teacher education** We appreciate the suggestion to move beyond abstraction by indicating how PIK can be used as a diagnostic lens in teacher education programmes. We addressed this by adding an explicit “use-case” in the conclusions.

- **Added “PIK as diagnostic tool in teacher education” (Section 7.2):** We describe how programmes can collect evidence across three complementary layers—(i) professional knowledge and competencies, (ii) identity indicators, and (iii) bridging constructs—and how these sources can be used formatively to identify alignments and tensions and guide targeted interventions (e.g., mentoring, guided rehearsal, design-based cycles).

This addition is meant to clarify how the model can be operationalised in realistic teacher education settings using low-burden artefacts commonly employed in programmes (e.g., annotated lesson plans, video-based noticing tasks, structured reflective prompts, portfolio entries).   Finally, we would like to thank you again for your supportive evaluation (comprehensiveness, citation support, accessibility, and appropriateness of conclusions). Your recommendations helped us improve the manuscript’s methodological transparency and strengthen the connection between the integrative synthesis and the model’s structure and potential use in teacher education. Sincerely,

 José Piñero (on behalf of the authors)

# Reviewer report: Reviewer response for version 2

**DOI:** 10.21956/openreseurope.24907.r69142

**Published:** 2026-03-11

**Authors:** Carlit Casey Tibane

**Affiliations:** 1 Tshwane University of Technology, Pretoria, Gauteng, South Africa

In a careful review, the authors have improved the paper with minor acceptable issues, therefore the final decision is Accept the paper at its current state.

Are all factual statements correct and adequately supported by citations?

Partly

Is the review written in accessible language?

Partly

Are the conclusions drawn appropriate in the context of the current research literature?

Yes

Is the topic of the review discussed comprehensively in the context of the current literature?

No

Reviewer Expertise:

NA

I confirm that I have read this submission and believe that I have an appropriate level of expertise to confirm that it is of an acceptable scientific standard.

# Reviewer report: Reviewer response for version 1

**DOI:** 10.21956/openreseurope.22831.r66613

**Published:** 2026-01-21

**Authors:** Carlit Casey Tibane

**Affiliations:** 1 Tshwane University of Technology, Pretoria, Gauteng, South Africa

The manuscript presents a theoretically robust and well-structured review that engages substantively with contemporary literature on mathematics teacher professional knowledge and identity. The topic is discussed in a largely comprehensive manner within a clearly defined theoretical boundary, offering an integrative synthesis rather than an exhaustive mapping of all possible perspectives. Factual statements are generally accurate and supported by relevant and up-to-date citations; however, some higher-level interpretive claims and model-specific propositions rely primarily on authorial synthesis and would benefit from clearer signaling as conceptual in nature or from the addition of supporting references where available. The review is written in clear and coherent academic language appropriate for a specialist readership, although its conceptual density and extensive use of specialized terminology may limit accessibility for broader audiences. To strengthen the manuscript further, the authors are encouraged to clarify the methodological positioning of the review as a theoretical or integrative synthesis, explicitly delimit the scope of the literature considered, and include brief signposting or summarizing statements in longer sections to enhance readability.

 The article presents a strong and credible theoretical contribution, and its central arguments and conclusions are largely justified and well aligned with the current literature. However, it would benefit from targeted revisions to strengthen methodological transparency, clarify the conceptual scope of the review, and improve accessibility for a broader readership.

Are all factual statements correct and adequately supported by citations?

Partly

Is the review written in accessible language?

Partly

Are the conclusions drawn appropriate in the context of the current research literature?

Yes

Is the topic of the review discussed comprehensively in the context of the current literature?

No

Reviewer Expertise:

NA

I confirm that I have read this submission and believe that I have an appropriate level of expertise to confirm that it is of an acceptable scientific standard, however I have significant reservations, as outlined above.

# Response

**Published:** 2026-01-28

**Authors:** José Carlos Piñero Charlo

Dear Reviewer,

 We sincerely thank you for your words and for the time and care you devoted to reading our manuscript and for providing detailed, constructive feedback. Let me respond, by using your letter as a template, to each requirement and evaluative remark. For ease of review, I’ve indicated where changes were implemented by referencing the relevant sections of the revised manuscript. I’ll proceed to address each requirement.

**1. Methodological positioning of the review and support for factual statements**

 We fully agree. In the revised manuscript we have clarified, at a higher level of prominence, that the paper is an *integrative theoretical synthesis* rather than an exhaustive mapping or a formal “systematic review” in the strict PRISMA sense. As follows:

- **Abstract (methodological positioning):** We added an explicit statement positioning the article as an integrative theoretical synthesis and clarifying that it combines a structured search and thematic synthesis without claiming exhaustive coverage.
- **Methodological approach (early signposting):** We added a short “positioning” subsection that explains the rationale for an integrative synthesis in relation to model construction and conceptual re-organization.
- **Supporting methodological references:** To avoid reliance on authorial assertion, we incorporated established methodological sources on integrative/theoretical reviews and review typologies, which justify the approach adopted.

Because the main contribution of the manuscript is conceptual (i.e., the construction and articulation of an interdependent model linking teacher knowledge and teacher identity), an integrative theoretical synthesis is the most appropriate methodological form. The revised wording is intended to align the reader’s expectations with the actual goals and procedures of the review.

 Furthermore, Reviewer #1 (Dr. Carlit Casey) states that the manuscript could benefit from clearer signaling as conceptual in nature or from additional references where available. This is because of a couple of ungrounded statements. So, we agree and have implemented two complementary improvements (a) and (b):

**(a) Clearer signaling of epistemic status**

- **Epistemic-status paragraph for the model:** We added an explicit statement clarifying that the relational links within the model are **conceptual propositions** derived from comparative synthesis, intended as **testable hypotheses** rather than empirically established causal claims.

**(b) Additional supporting references where feasible**

- For claims about the integration of disciplinary, didactic, and contextual knowledge—and the role of situated cognitive skills in practice—we reinforced citation support with widely recognized international and Spanish scholarship.
- We also reviewed several interpretive passages and refined the language to avoid over-strong generalizations (e.g., replacing categorical formulations with appropriately modalized phrasing where the claim is interpretive).

These changes maintain the manuscript’s theoretical contribution while aligning the presentation of claims with standard scholarly expectations: statements are supported with citations, and conceptual claims are transparently framed as propositions emerging from synthesis.

**2. Delimitation of the Scope and accessibility**

 R#1 aims for clarification of the scope, so we have strengthened the manuscript’s scope statement and made the boundaries of the corpus more explicit and reader-visible:

- **Databases, timeframe, and language:** We state clearly that the corpus was delimited to peer-reviewed journal articles indexed in Scopus and Web of Science, published in the 2015–2025 timeframe, and written in English or Spanish.
- **Search strategy and selection:** We clarify the search strings, screening process, and inclusion/exclusion criteria, and we report the initial records and final corpus used for synthesis.
- **Rationale for delimitations:** We explicitly justify why these boundaries were necessary to maintain conceptual coherence and ensure feasibility for an integrative synthesis aimed at model construction.

These additions increase methodological transparency while preserving the manuscript’s theoretical intent. They also directly address the concern that the review might otherwise be read as claiming a level of exhaustiveness that was not intended.

 Furthermore, we’ve also addressed the “conceptual density” issue by improving readability without diluting the specialist-level rigor:

- **Signposting:** We added short roadmap sentences at the start of selected longer sections to orient the reader to the section’s function in the overall argument.
- **Summarizing closures:** We inserted brief “In sum…” statements at the end of longer conceptual sections to consolidate the key takeaways before transitioning.
- **Terminology management:** Where possible, we reduced repeated technical phrasing and clarified transitions between knowledge-focused and identity-focused constructs to support readers who may be more familiar with one literature than the other.

These revisions are intended to preserve conceptual precision while making the synthesis easier to navigate for a broader readership across the mathematics education community.

**3. About the assessment of the discussion in the context of the literature**

 We acknowledge this evaluation and have revised the manuscript to ensure that the *intended* goal is explicit: the article is an **integrative synthesis** aimed at conceptual model construction rather than an exhaustive mapping of all perspectives. To address the underlying concern, we (i) strengthened the scope statement and delimitations, and (ii) clarified the methodological positioning so that the manuscript is assessed against the appropriate standard for integrative theoretical reviews.

**4. About the assessment of the support on citations**

 Some statements were not supported by citations. We addressed this by (i) strengthening citation support for selected high-level claims (particularly those concerning integrated professional profiles and situated cognitive skills), and (ii) explicitly labeling model links as conceptual propositions where the claim is interpretive. We also revised language in a small number of passages to avoid any unintended implication of empirically established causality.

**5. About the accessibility**

 We improved accessibility through signposting and summary statements, and by tightening transitions and reducing unnecessary repetition of specialized terminology, while retaining the precision required for a specialist audience.

**Final considerations**

 We appreciate this positive evaluation. In the revised manuscript, we preserved the core conclusions while strengthening the methodological framing and evidentiary signaling that support the conclusions’ validity.

 Finally, we’ve addressed additional revision (not explicitly requested by R#1, but implemented for coherence), by renaming and checking the consistency of the model acronym -which remained with the Spanish-like acronym-. So, during revision, we also refined the model’s acronym (now consistently presented as **PIK**) and ensured that all occurrences in the text, headings, and figure captions match the new naming. To mi opinion, this change improves clarity and reduces potential confusion by ensuring consistent referencing across the manuscript and its visual elements.

 Once again, we are grateful for your thoughtful review. Your feedback has materially strengthened the manuscript’s methodological transparency, conceptual clarity, and overall readability. We hope the revised version satisfactorily addresses your concerns, and we would welcome any further suggestions you may have.

 Sincerely,

 José Piñero
